# The Natural and Clinical History of Plague: From the Ancient Pandemics to Modern Insights

**DOI:** 10.3390/microorganisms12010146

**Published:** 2024-01-11

**Authors:** Antoni Bennasar-Figueras

**Affiliations:** 1 Microbiologia—Departament de Biologia, Universitat de les Illes Balears (UIB), Campus UIB, Carretera de Valldemossa, Km 7.5, 07122 Palma de Mallorca, Spain; toni.bennasar@uib.es; Tel.: +34-971172778; 2 Facultat de Medicina, Hospital Universitari Son Espases (HUSE), Universitat de les Illes Balears (UIB), Carretera de Valldemossa, 79, 07122 Palma de Mallorca, Spain

**Keywords:** plague, *Yersinia pestis*, ancient DNA, bioinformatics

## Abstract

The human pathogen *Yersinia pestis* is responsible for bubonic, septicemic, and pneumonic plague. A deeply comprehensive overview of its historical context, bacteriological characteristics, genomic analysis based on ancient DNA (aDNA) and modern strains, and its impact on historical and actual human populations, is explored. The results from multiple studies have been synthesized to investigate the origins of plague, its transmission, and effects on different populations. Additionally, molecular interactions of *Y. pestis*, from its evolutionary origins to its adaptation to flea-born transmission, and its impact on human and wild populations are considered. The characteristic combinations of aDNA patterns, which plays a decisive role in the reconstruction and analysis of ancient genomes, are reviewed. Bioinformatics is fundamental in identifying specific *Y. pestis* lineages, and automated pipelines are among the valuable tools in implementing such studies. Plague, which remains among human history’s most lethal infectious diseases, but also other zoonotic diseases, requires the continuous investigation of plague topics. This can be achieved by improving molecular and genetic screening of animal populations, identifying ecological and social determinants of outbreaks, increasing interdisciplinary collaborations among scientists and public healthcare providers, and continued research into the characterization, diagnosis, and treatment of these diseases.

## 1. Introduction

The bacterium responsible for the plague, *Yersinia pestis*, has profoundly impacted human history, emerging as one of the most destructive diseases, responsible for several pandemics and millions of deaths. This infectious disease has caused the greatest terror worldwide, particularly through the three major plague pandemics. The Book of Samuel in the Old Testament of the Bible (written during 630–540 BCE), in an episode from chapter 5, describes the capture of the Ark of the Covenant by the Philistines and an epidemic of tumors and death occurring in the city of Ashdod. Several painters have been inspired by this biblical scene, although probably one of the best worldwide know is “*The Plague at Ashdod*” (1630) (Louvre Museum, Paris, France) by Nicolas Poussin. This famous painting depicts rats running between buildings and dead and dying bodies, in theory, in the midst of a plague [1]. Although some authors have questioned the relationship between the plague described in the biblical work and the bubonic plague, fossilized remains of fleas *Xenopsylla cheopis* and black rats *Rattus* have been found in the Egyptian Nile Valley, indicating that these plague carriers were already in the Middle East in 1350 BCE [1].

A comprehensive review of the natural and clinical history of the plague caused by *Y. pestis* needs to cover various aspects that, together with the main structure of the review, are next briefly described and afterwards detailed through each section of the present review. Effectively, initially, a detailed examination of the history of plague pandemics, tracing the impact of the disease from ancient times to the present, is analyzed in the Introduction section. The ancient DNA (aDNA) studies section aims to review the presence of *Y. pestis* in historical populations and the actual molecular diagnoses, genomic and bioinformatic approaches to help exploring the pathogen’s evolutionary history, comparing ancient with modern strains described under the next section related to evolutionary origins. A microorganism profile section follows to review the advances produced in its characterization, from the discovery of the bubonic plague agent to the actual nomenclature changes, pathogenesis, virulence factors, and adaptations. The epidemiology section covers information related to the analysis of the distribution, determinants, and control of plague throughout history, focusing on patterns of transmission and public health implications. A section on the diagnosis, treatment, and prevention of plague outlines the methods for identifying *Y. pestis* infections, the antibiotic treatments, and highlights the importance of public health measures such as rodent control and prevention of plague outbreaks. It also addresses the utility of point-of-care testing in areas lacking advanced laboratory facilities. In the prevention context, the vaccine subject is reviewed in the next section to evaluate recent advancements in plague vaccines, including oral, bacteriophage, and recombinant vaccines, and to discuss the ongoing challenges in vaccine development and the need for further research in this field. Finally, the “Things to Do in Plague Research” section aims to identify gaps in current knowledge and suggest future research directions to better understand and combat plague.

### 1.1. The First Pandemic—The Plague of Justinian

The first described pandemic, known as the Plague of Justinian, occurred in the 6th century (year 541) in the Egyptian port of Pelusium, according to the most plausible hypothesis, and continued in 14 to 21 additional waves until 750/767, collectively referred to as the “first pandemic [2,3]. The first wave was named the “plague of Justinian” after the emperor of the Roman Empire of the Orient, Justinian I (527–565), and initially spread around the Mediterranean Basin between 541 and 544 [3,4]. Once this pandemic finished in the mid-700s, a considerable percentage of the population of these countries had succumbed to the plague [5]. Although, the first pandemic has traditionally been thought to be responsible for tens of millions of deaths, the exact number of deaths remains a subject of discussion, and some studies suggest that the mortality estimates have been exaggerated and are not supported by the existing evidence [4].

### 1.2. The Second Pandemic—Black Death

The second pandemic, in the 14th century, which started in the 1320s, again reached catastrophic proportions, perhaps due to a vicious cycle of deteriorating social conditions, which caused rats to approach people. This pandemic, known as the Black Death due to the intense cyanosis of the dying, killed approximately 1/4 of the European population and spread to the Middle East and the Far East. With improvements in living conditions, the disease receded in Europe, but serious epidemics emerged in other countries.

The Black Death in the 14th Century (AD 1346–1353), plague caused by *Y. pestis*, stands as the most widespread and lethal pandemics of this bacterium in human history, causing more than 50 million deaths in Europe according to the WHO [6], or 25 million (30% to 40% of the population) according to Glatter and Finkelman [7]. This is an example of those data and the precise circumstances leading to the introduction of *Y. pestis* into human populations causing the second plague pandemic, and the specific time and location of its emergence are not definitively established. However, various hypotheses have been proposed to explore these events. Although the exact origin is still unknown, regarding the original geographic source of the Black Death, it seems that it emerged in the 14th century in the Eurasian Steppes, ranging from Western Europe to Eastern Asia. This is supported by a synthesis of archaeological, historical, and ancient genomic DNA data in central Eurasia. This was demonstrated by ancient DNA data retrieved from seven individuals exhumed from two cemeteries near Issyk-Kul in Kyrgyzstan, two of which were complete genomes of ancient *Y. pestis* [8]. By contrast, the analysis of historical, genetic, and ecological evidence has led to the hypothesis that the emergence of *Y. pestis* occurred over a century before the onset of the Black Death. The suggested model posits that this early diversification was influenced by human interactions and correlated with the territorial expansions of the Mongol Empire across Eurasia, ultimately extending to the Black Sea in the early 13th century [8]. Italian merchants then brought it to the Mediterranean, arriving to Messina in Sicily via Genoese ships carrying flea-laden rats in October 1347 and, from there, the plague spread to almost every corner of Europe, the Middle East, and North Africa. In Europe, the plague continued its way around the West, first to France and Spain by 1348 and then reaching Germany, Switzerland, and Austria, and in 1349, it reached Scandinavia via Northern England moving into 1350 [7,9,10,11]. Traveling along the Baltic coast, it arrived in Russia by 1351 [12]. While some regions in Europe remained untouched by the plague for decades, others experienced a delayed impact, e.g., Iceland was unaffected until 1402, at which point the plague caused a substantial decline in its population, with some authors estimating a mortality rate of 50–60% of the population within just two years [13].

The impact of the Black Death in Europe was deep, and precise death tolls remain uncertain about the exact numbers due to contradictions between different sources. In some of them, the death tolls even surpass the total population estimates at the time. The pandemic, estimated to have lasted eight years, is assumed to have resulted in the demise of up to 60% of the Western Eurasian population [8,14,15]. This resulted in notable demographic and socioeconomic repercussions across all impacted regions, with European historical documentation standing out as the most deeply investigated repository to date [8]. The effects were particularly severe in major harbor cities and urban areas across Europe, leading to devastating consequences during the epidemic’s peak in cities, like Venice, Florence, and Siena [16].

**Figure 1:**
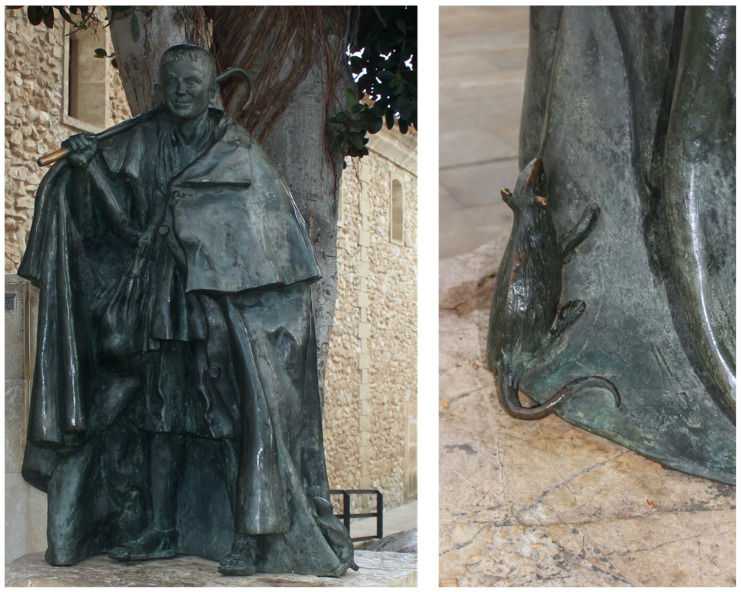
The Shepherd of Son Servera. In 1820, an outbreak of plague occurred in the eastern part of the island of Majorca, resulting in significant mortality and demographic effects in the area. Physicians actively investigated the entry and transmission of the disease at the time. The events inspired imaginative literary interpretations that, while diverging from reality, contributed to the emergence of a legend. Interestingly, this legend, which is now widely accepted as factual, revolves around a mysterious figure -an unnamed young shepherd. According to the legend, this enigmatic personage supposedly contracted the plague by handling a cape abandoned in a tomb where the body of a subject who succumbed to the plague disease would have been buried, without the knowledge or approval of local authorities. The supposed plagued, hypothetical starting point and source of the epidemic would have been disembarked from a ship without an identified name, with origin attributed from Tangier [22].

Continuing to explore the dynamics of the disease, plague persisted in Europe for more than five centuries following the Black Death, although changing its dynamics by the 17th century, contributing to a gradual decline in Western Europe over the ensuing two centuries [17]. Interestingly, a significant shift in plague dynamics occurred around the mid-17th century, with merely one reported continental-scale epidemic in Western Europe during the early 1700s [17,18,19]. Although improved public health measures and medical understanding helped to prevent large-scale occurrences of the disease, localized outbreaks of plague still occurred in Europe until the early 19th century, like the bubonic plague that reached Malta from Alexandria on 29 March 1813 [20] and Mallorca (1820) [21]. The bubonic plague in Mallorca in 1820 is known as the “latest plague in Europe of the 19th century”, and the east of Mallorca was affected by a devastating outbreak of plague that apparently arrived by sea and landed merchandise on the coast of Son Servera (Figure 1). The first victim, on May 9 in Son Servera, was followed by up to 2419 dead (33% of the total of inhabitants before plague) in the affected areas (Son Servera, Artá, and Capdepera).

### 1.3. The Third Plague Pandemic

The Third Plague Pandemic, which began in China in the 1850s, was a historic turning point in our knowledge of the associated pathogen. Initially confined to Yunnan, socioeconomic factors, such as the Panthay Rebellion, triggered a diaspora of refugees, spreading the disease throughout China [23]. Subsequently, British merchants transported the pathogen from Hong Kong to other empire territories, with India suffering the highest losses due to plague [7,9,14,23]. During this pandemic, scientific advancements in Hong Kong led to the identification of the plague agent, the *Yersinia pestis* bacterium [24], and the elucidation of the relationship between rats, fleas, and disease transmission [25,26,27].

This pandemic persisted across all inhabited continents until the second half of the 20th century, although the plague declined worldwide in the first half, with occasional outbreaks occurring in Asia and Africa and sporadic cases in South America and the American Southwest [16]; since 1960, there has been a gradual increase, with a recent resurgence (1972–1975) on several continents. A total of 2737 cases were reported worldwide in 1974 (including 8 in the US), with 164 deaths (Morbidity and Mortality Weekly Report from CDC, 1976). In 1977, 18 cases were reported in the United States [14]. The contemporary occurrences of plague outbreaks were of 3248 cases and 584 deaths reported globally between 2010 and 2015 [28]. In August 2015, two unrelated teenagers visiting Yosemite National Park in California contracted plague in separate incidents, likely from infected squirrels. Also, local bears exhibited antibodies against *Y. pestis* [29]. A death case from bubonic plague in July 2020 of a teenage male in Mongolia after consuming an infected marmot was reported [30]. Whereas current plague outbreaks are manageable with antibiotics, the inherent presence of *Y. pestis* in populations of wild rodents across various regions (including eastern Europe, Asia, Africa, and the Americas) makes impossible its complete eradication from nature.

During ancient times, *Y. pestis* was the causative agent of extensive pandemics of plague, often following the pathways of human migration. The dissemination of plague was intricately linked to various human activities, including maritime trade and interactions along the Silk Road, facilitating the transportation of fleas associated with live rodents or commodities that contributed to the spread of the plague [31,32]. Nevertheless, in contemporary times, occurrences of human plague cases are predominantly confined to localized epidemics commonly termed as ‘plague foci’ [14,15,16]. Natural plague foci exhibit a widespread distribution across Asia, Eurasia, Africa, and the greater American region. From 2001 onwards, the World Health Organization (WHO) has documented 14 major outbreaks, primarily originating from Africa and Asia (https://www.who.int/news-room/fact-sheets/detail/plague, accessed on 2 January 2024). Consequently, *Y. pestis* is still a public health threat today, causing sporadic infections and occasional local epidemics [4,8,12,33,34,35,36].

## 2. Ancient DNA (aDNA) Studies

The main objective of studying *Y. pestis* through ancient DNA (aDNA) is to confirm its presence in historical populations and provide a direct molecular diagnosis of ancient septicemic plague epidemics. The study of *Y. pestis* aDNA allows for the investigation of the evolutionary history of the pathogen, the routes of historical pandemics, and the genetic changes that have occurred over time. This kind of research aims to identify specific genetic variations, which involves identifying distinct *Y. pestis* clones responsible for these epidemics, enhancing understanding of how these devastating events spread across populations. The assessment of the *Y. pestis* evolutionary patterns includes comparing ancient strains with modern ones, providing insights into the pathogen adaptation and evolution over the centuries. This analysis, beyond historical perspectives, emphasizes the examination of genetic makeup in ancient and contemporary strains, allowing scientists to trace the development of the bacterium and understand how it has adapted to changing environments and hosts throughout millennia.

The studies based on aDNA, and specifically on the pathogen *Y. pestis*, are constantly refining a number of critical aspects to enhance the reliability of results [37]. These include the sample preparation, DNA extraction, amplification, library preparation, and analysis of aDNA, which is frequently fragmented and chemically altered. Given the challenges posed by the degradation and modification of aDNA over time, refining these methods is crucial for ensuring the accuracy of research findings and addressing contamination concerns in studies involving ancient materials. Although various methods and protocols exist, the use of validated assays, avoiding contamination, and assessing DNA preservation are crucial.

A general consensus protocol for the detection of *Y pestis* in aDNA could be envisioned following a series of steps that ensure the authenticity and lack of contamination of the DNA samples. An optimized sample collection consists of obtaining skeletal remains from pandemic sites or other relevant archaeological contexts, preferably teeth or bones with high preservation potential. Most such studies focus on obtaining direct evidence of past septicemic infections by identifying *Y. pestis* aDNA preserved in the dental pulp of human remains. Dental pulp is a particularly reliable source for such studies due to its naturally sterile environment and its ability to remain intact over long periods. In order to prevent contamination, strict aseptic techniques during sample collection and preparation should be followed to prevent modern DNA contamination, using dedicated ancient DNA facilities if possible [3,37,38]. Depending on if dental pulp or bone samples are used, established protocols for the extraction of aDNA exist [37,39]. The dental pulp samples are usually prepared by sectioning teeth at the dentin–enamel junction and using rounded dental drill bits to obtain tooth powder, which is used for DNA extractions following optimized protocols for recovering short fragments of DNA [8]. For bone samples, the protocols include the removal of surface contaminants and the pulverization of bone under sterile conditions. Quantitative PCR assays on specific genes are usually employed for the initial screening of *Y. pestis* DNA. Next, target enrichment approaches are employed to capture and sequence, via next-generation sequencing (NGS), specific fragments of *Y. pestis* DNA [37,39].

Finally, various genomic analyses can be performed on billions of sequence reads, including full plasmid reconstruction of virulence-associated plasmids like pPCP1 [14,40]. This enables a deeper understanding of the pathogen’s virulence factors and historical strains. Additionally, the detection of Single Nucleotide Polymorphisms (SNPs) [41] facilitates the assessment of genetic diversity and evolutionary history, aiding in the identification of specific *Y. pestis* lineages [12,32,34,36]. Genome scanning is also employed to identify genomic regions of interest, such as deletions affecting virulence factors [34].

For these purposes, bioinformatics plays a decisive role in the analysis and reconstruction of aDNA. Due to the unique characteristics of aDNA, such as low endogenous DNA content, short fragment lengths, and characteristic misincorporation patterns due to damage, specialized bioinformatics methods are required to successfully analyze aDNA [31,39,40,42,43]. The application of standard alignment methods with default parameters is often not suitable for aDNA, requiring the development of tailored approaches to address these challenges. Effectively, the computational reconstruction of ancient genomes from High-Throughput Sequencing (HTS) data typically involves short read alignment methods like BWA [44], along with standard analysis toolboxes such as SAMtools [41,45] or the Genome Analysis Toolkit (GATK) [46]. However, the default parameters of modern alignment methods may not be suitable for aDNA, requiring adjustments to account for the specific damage patterns (which is a crucial step in authenticating the ancient origin of the DNA samples) and common fragmentation in ancient samples.

**Figure 2:**
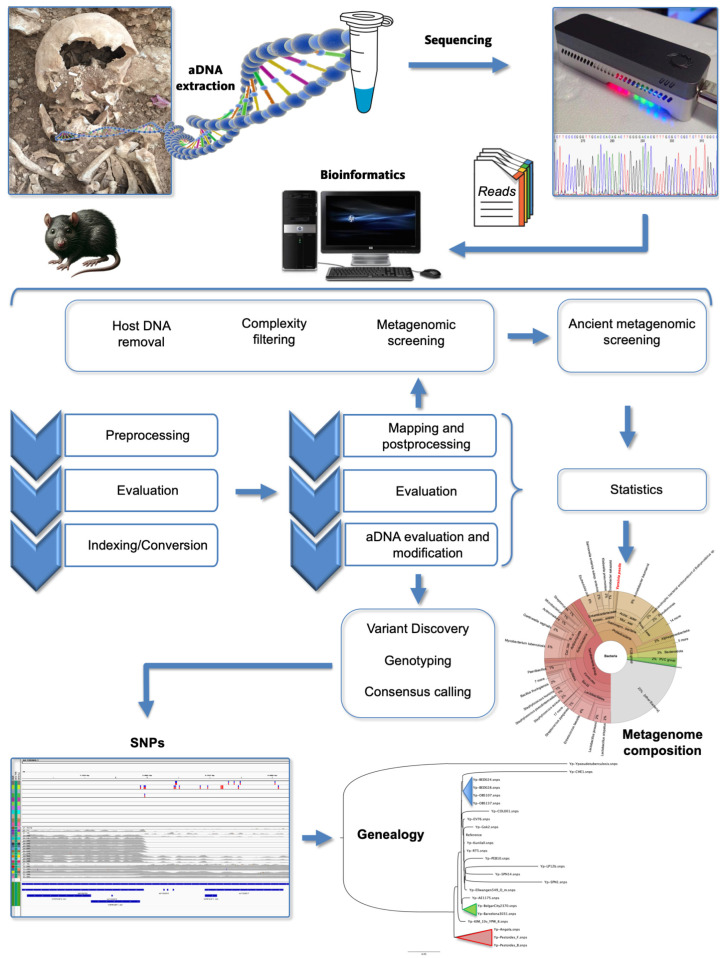
General workflow of the in silico analysis for ancient DNA (aDNA). The bioinformatics pipelines processing genomic NGS sequencing data, focussed on aDNA data, adopt similar strategies to integrate conventional tools applied in the processing of sequencing data (e.g., Illumina HTS short-read). The workflows commonly encompass tasks such as pre-processing the raw data inputs (usually reads in FASTQ format) or preprocessed alignment inputs, sequencing quality assessment, removal of sequencing adapters and merging of paired-end reads, mapping to a reference genome, aDNA-specific quality-control (i.e., including the assessment for damage patterns such as nucleotide misincorporation and fragmentation patterns), genotyping and metagenomic screening for specific microbes or even microbiome composition. Optimized samples from pandemic sites are well-preserved skeletal remains, preferably teeth or bones, to detect *Y. pestis* in ancient DNA. The dental pulp is a particularly reliable source due to its naturally sterile environment and to remain intact over long periods. Details for specific pipelines are described in the text and references [47,48,49].

Bioinformatics tools and pipelines, such as PALEOMIX [47], EAGER [48], and HOPS [49], have been specifically designed to process and analyze aDNA data, enabling researchers to authenticate ancient samples, identify species, and reconstruct genomic sequences from the past. Therefore, bioinformatics is not only important but essential for the accurate analysis and interpretation of aDNA data (Figure 2). The PALEOMIX pipeline offers toolkits for mapping reads, genotyping, and taxonomic as well as metagenomic profiling of aDNA samples. PALEOMIX is designed to be user-friendly and is capable of handling the complexities and challenges associated with aDNA datasets, such as contamination, short fragment lengths, and post-mortem DNA damage. Another comprehensive pipeline designed for aDNA data is EAGER (Efficient Ancient Genome Reconstruction), which includes modules for preprocessing, read mapping, damage pattern analysis, genotyping using GATK, and contamination estimation. EAGER provides a graphical user interface that simplifies the configuration of the analysis, making it accessible to users who may not be familiar with command-line tools. EAGER also incorporates new tools for efficient adapter clipping, read merging, and de-duplication, which are essential steps in the processing of aDNA sequencing data. The pipeline code is available at https://github.com/nf-core/eager (accessed on 2 January 2024) and archived with Zenodo, including the last up-to-date version, 2.5.0, published in November 2023, under the DOI 10.5281/zenodo.1465061 (accessed on 2 January 2024). Another valuable tool in the field of paleogenomics and ancient disease research is HOPS (Heuristic Operational Pipeline System), an automated screening pipeline designed for the analysis of aDNA, particularly for detecting and authenticating pathogen DNA in archaeological remains. It is tailored to address the challenges of identifying ancient pathogens within metagenomic datasets. HOPS stands out for its specificity and sensitivity, being able to detect pathogen DNA even when it is present in low abundance. It also includes an evaluation of DNA damage patterns.

## 3. Evolutionary Origins

The genetic diversity of *Y. pestis* during pandemics has been explored through the reconstruction of ancient genomes, which offer insights on the bacterium’s evolutionary path and the impact of historical diseases on human evolution. The high-throughput genome sequencing analysis of ancient human remains confirms that the pathogen responsible for the three pandemics is *Y. pestis* [37]. Moreover, the recovery of *Y. pestis* DNA from prehistoric teeth also revealed the branching of independent lineages across Eurasia during the Neolithic period [32,36]. This discovery has allowed for a more accurate estimate of the evolutionary divergence of *Y. pestis* and *Y. pseudotuberculosis*, which occurred relatively recently, with estimates ranging from 2600 to 28,000 years ago [35]. Other comparative genomics and population genetic research suggest that *Y. pestis* diverged from *Y. pseudotuberculosis* relatively recently, between 1500 and 6400 years ago. [31,50,51,52,53]. The identification of *Y. pestis* in the reconstructed genome of a 5000-year-old Latvian hunter–gatherer may represent an early instance of septicemic plague resulting from zoonotic transmission. This finding possibly corresponds to one of the first strains in a series of ancient strains that evolved shortly after *Y. pestis* diverged from its predecessor, *Y. pseudotuberculosis*, around 7000 years ago [54].

**Figure 3:**
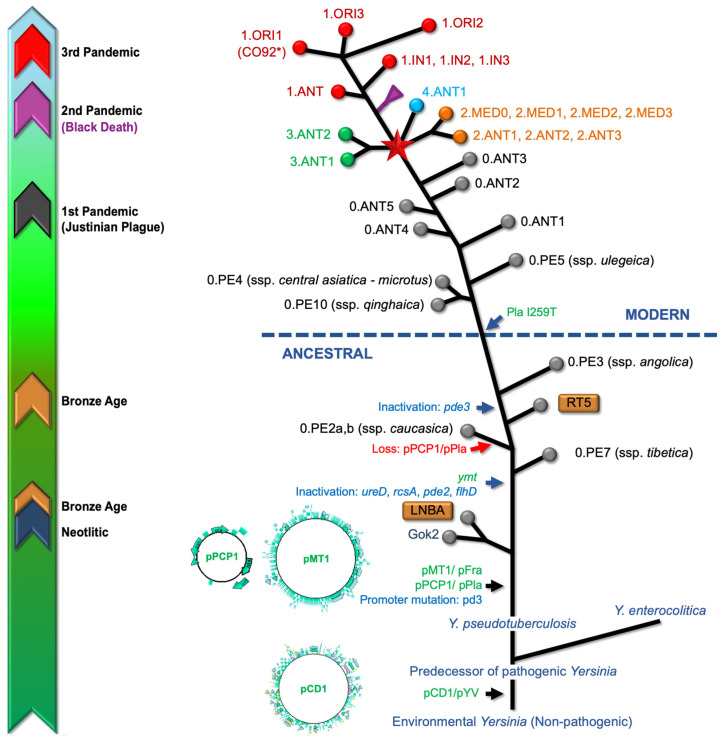
Graphic representation of the *Yersinia pestis* genealogy based on the consensus information recompiled from several sources [56,59]. The timely historical guide (left) refers to registered periods for aDNA specimens and recorded plague pandemics. The enteropathogenic *Yersinia* species, including *Y. enterocolitica*, *Y. pseudotuberculosis*, and *Y. pestis* harbors the pCD1/pYV, an important 70-kb virulence plasmid that plays a key role in facilitating infection, circumventing host defense mechanisms [60]. The acquisition of this plasmid implied a change from an hypothetical non-pathogenic environmental *Yersinia* into a predecessor of the pathogenic members of this species [55,60,61]. The acquisition of two virulence-associated plasmids (pMT1, pPCP1) and chromosome rearrangement processes seems that turned *Y. pseudotuberculosis* into *Y. pestis* [51,60]. Additional significant genetic events have been observed throughout the evolutionary trajectory of *Y. pestis*, including the acquisition of the *Yersinia* murine toxin (*ymt*) gene, within the pMT1 plasmid and the inactivation of genes associated with virulence. The extinct Neolithic (Gok2) and Bronze Age lineages (LNBA and RT5) are included [62]). Genomic comparative analyses indicate that the I259 Pla isoform is an ancient variant, so the acquisition of the I259T mutation in the plasminogen activator, occurring after the 0.PE3 (ssp. *angolica*) strain, is a distinctive feature characterizing the so-called modern strains [56,59,63]. The node, indicated by a red star, represents the so-called “Big Bang” event in the evolutionary history of *Y. pestis*; which led to the emergence of Branches 1 to 4 and significantly contributed to the existing strain diversity, including lineages associated with the Modern plague pandemic [31,64]. Abbreviations: ANT (Antiqua), MED (Medievalis), ORI (Orientalis), IN (Intermediate), PE (Pestoides) [65]. Several branches have been collapsed for clarity, including the Black Death and Intermediate strains clusters of Branch 1 and Branch 2 (Antiqua and Medievalis). Colors codes used for circles at the branch ends are (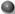) Branch 0, (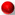) Branch 1, (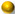) Branch 2, (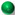) Branch 3, and (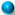) Branch 4. Branch length does not represent the evolutionary time. * The genome of *Y. pestis*, strain CO92 (including plasmids) is usually used as reference for reads mapping from aDNA [64,66].

Despite sharing a high degree of genetic similarity with *Y. pseudotuberculosis*, *Y. pestis* has undergone key genetic changes (Figure 3), including the acquisition of genes and plasmids that facilitate flea-borne transmission, a critical factor influencing widespread pandemics and the subsequent emergence of bubonic plague, enabling it to cause rapid and often fatal systemic infections in its hosts [53,55,56]. This was an evolutionary shift from an enteric to a flea-transmitted life cycle, with the ability to cause pneumonic plague through the acquisition of the Pla protease gene, indicating that *Y. pestis* could infect the lungs soon after its divergence from its progenitor species [53,55], representing a significant difference from the lifestyle of its progenitor species, a more prevalent and environmental stress-tolerant, but less pathogenic, enteric bacteria [35]. In this context, some evidence challenges the notion that the Pla protease gene alone determines the ability of *Y. pestis* to cause pneumonic plague. Effectively, it has been demonstrated that Pla-deficient strains like Pestoides F induce pneumonic plague, suggesting the existence of compensatory virulence factors [57]. Furthermore, Pla-lacking strains from common voles, despite the absence of the Pla protease, were capable of causing pneumonic plague that was transmitted from person to person via aerosols, without the involvement of a flea vector [58].

The varied mutation rate during its evolution suggests that *Y. pestis* did not strictly adhere to a constant evolutionary clock, leading to its current status as a clonally expanded, genomically degenerating variant of *Y. pseudotuberculosis* (Figure 3) [53]. The two newly acquired plasmids (pMT1 and pPCP1), together with the plasmid directly inherited from *Y. pseudotuberculosis* (pCD1), significantly contribute to the pathogenicity of *Y. pestis*, together with numerous chromosomal loci, for example, *pgm*, carrying a highly pathogenic island with iron acquisition functions and biofilm formation [67,68]. The plasmid pCD1 encodes for a type III secretion system (T3SS) that exhibits a needle-like configuration on the bacterial surface. This structure facilitates the injection of toxic *Yersinia* outer proteins (Yops) into host cells upon direct interaction between the pathogen and the host cells [67,69]. Yops, in turn, can impede the action of the host’s innate immunity and dismantle cellular structures, thereby playing critical roles in the development of plague disease. Regarding *Y. pestis*, the plasmid pMT1 is responsible for encoding the fraction 1 (F1) capsular antigen. This antigen is widely used as a vaccine component and serves as a diagnostic target. Additionally, pMT1 encodes the *Yersinia* murine toxin (Ymt), a phospholipase D [70,71], which is not required for virulence but is an essential factor for the survival of the bacteria within the flea gut [70,71]. The plasmid pPCP1 is responsible for encoding a proteinase, plasminogen activator (Pla), which is essential in facilitating bacterial invasion into host tissues. The adhesion and proteolytic capabilities of Pla are used by *Y. pestis* to control both the fibrinolytic cascade and the immune system, leading to the development of bacteremia, necessary for pathogen transmission through fleabites or aerosols [63,72].

Conventionally, based on subtle phenotypic distinctions, such as the nitrate-to-nitrite conversion and glycerol fermentation, *Y. pestis* has been divided into three biovars (Antiqua, Medievalis, and Orientalis) [51,73,74]. A fourth biotype, Microtus, describes Medievalis isolates lacking arabinose fermentation [74]. The epidemiological findings and historical records regarding the biotype of the plague bacterium from different eras suggest that the biotypes Antigua, Medievalis, and Orientalis have historically originated in sequence [51,75], i.e., that biovar Antiqua, with strains presently contributing to foci in Central Asia, Siberia, and the Russian Federation [65], is believed to have originated from bacteria responsible for the Justinian plague in the sixth century. In contrast, Medievalis, found in Central Asia, is thought to have descended from the bacteria that caused the second pandemic, the Black Death, in the 14th century. Bacteria epidemiologically linked to the third pandemic are all Orientalis, originating in Southern China in about 1890, and are currently widespread, being the predominant biotype today [73].

The whole-genome phylogenies based on the core genome SNP analysis defined a five-branch population structure for *Y. pestis* [31], and following previously established nomenclatures, the lineages are designated based on their phylogenetic branch (0–4) and the biovar abbreviations: ANT (biovar Antiqua), MED (Medievalis), ORI (Orientalis), IN (Intermediate), and PE (Pestoides, including Microtus isolates) [59]. Consequently, each branch represents different lineages and biovars of the bacterium, with Branch 0 being the root lineage and containing several ‘untypical’ groups such as the Pestoides and the biovar Microtus. Branches 1 through 4 emerged from a polytomy known as the “Big Bang” and are associated with the historically recorded plague pandemics [56,59].

The genomic findings regarding *Y. pestis* genomes from across Western Europe during the Plague of Justinian (541–750) show early diversification of the bacterium during the First Pandemic. This suggests that once *Y. pestis* was introduced to Europe, it quickly diversified into multiple lineages [34]. The results support the hypothesis that the bacterium initially entered through Eastern Europe, which aligns with historical accounts that the pandemic began in the Byzantine Empire and spread westward [12].

The phylogenetic analysis of SNPs across 14th century *Y. pestis* genomes provided insights into the genetic diversity and evolutionary history of *Y. pestis* during the Second Pandemic [8]. The analysis showed that the major genetic lineage related to the Black Death moved from Central Asia to Europe and persisted there for several centuries [56]. This lineage, part of Branch 1 of *Y. pestis*, is thought to have thrived in Europe during the late-medieval and possibly early modern periods, contributing to the waves of the Second Pandemic. The absence of genetic diversity in *Y. pestis* strains indicates a bottleneck or founder effect where a single or few lineages were responsible for this pandemic [12]. After the Black Death, the genomes show diversification of *Y. pestis* into multiple genetically distinct clades. This suggests the development of more than one disease reservoir in or near Europe, which could have implications for understanding the persistence and spread of the plague [12]. The emergence of four major lineages associated with strains that caused the Black Death in the 14th century, which are part of Branch 1 of *Y. pestis*, were dispersed in rodent foci in Eurasia, Africa, and the Americas, which is a significant finding in the study of *Y. pestis* and its historical pandemics [36,56]. Therefore, Branch 1 represents the most prevalent lineage of *Y. pestis*, currently thriving in natural plague foci in Asia, Africa, and America. It is also believed to have thrived in Europe during the late-medieval and possibly early modern periods [56,59].

The abovementioned hypothesis suggesting that the biotypes Antigua, Medievalis, and Orientalis have historically originated in sequence [51,75] faced challenges from several studies, which suggests a role of the Orientalis biotype in the three pandemics [73,74,76,77,78]. Analyzing the findings regarding the biotype of the plague bacterium from the different eras, the detected loss of a genomic region that includes virulence-related genes was associated with the late stages of the Black Death pandemic. This deletion was also identified in genomes connected with the First Pandemic, indicating a possible similar evolutionary trajectory of *Y. pestis* during both pandemics [12,34]. Further genotyping studies of *Yersinia pestis* in historical plague victims provide evidence for the long-term persistence of the bacterium in Europe from the 14th to the 17th century [79]. SNP-based analysis of aDNA from plague victims of the second plague pandemic and spanning over 300 years revealed that all positive individuals were identical in all 16 SNP positions tested [79]. This suggests that at least one genotype of *Y. pestis* introduced to Europe at the beginning of the Black Death from Asia persisted in Europe until the Thirty Years’ War (1618–1648) [79]. These findings challenge the previously held hypothesis that *Y. pestis* was continuously reintroduced to Europe from Central Asia in multiple waves during the Second Pandemic. Instead, the data indicate that there must have been a long-term persistence of the pathogen in Europe in a yet-unidentified reservoir host. This fact has significant implications for our understanding of the historical transmission routes of *Y. pestis* and the dynamics of plague outbreaks during that period [79].

The role of the Orientalis biotype in the three pandemics was demonstrated through a combination of genotyping studies using multiple spacer typing (MST) [78], analysis of specific gene deletions such as the 93 base pair deletion in the gene encoding glycerol-3-phosphate dehydrogenase (*glpD*) by sequencing from ancient dental pulp specimens, and determining the absence of glycerol fermentation in the Orientalis biotype [80,81], positive for the isolates of other biotypes [80]. These approaches associated the Orientalis biotype with the third pandemic, which began in the 19th century and includes the strains that spread around the world and are generally found in modern outbreaks [51]. The deletion in the *glpD* gene, which is involved in the metabolism of glycerol, is one of the genetic markers distinguishing the Orientalis biotype from earlier strains of *Y. pestis* and is thought to have implications for the bacterium’s virulence and its ability to survive in different environments and hosts [24,37]. The *Y. pestis* strains from the Justinian era (First Pandemic) and those from the medieval era (Second Pandemic, including the Black Death) differ in the presence of the *glpD* gene.

The molecular analysis of ancient human remains attributed to the First and Second pandemics showed that the sequences of certain genes, e.g., the *pla* plasmid gene and the chromosomal genes *rpoB* and *caf1,* were almost identical to those found in the Orientalis strain *Y. pestis* CO92 [77], suggesting that the Orientalis biotype was present during these earlier pandemics as well. Additionally, sequences related to the Orientalis biotype were found in ancient samples from verified plague sites associated to the Plague of Justinian and the Black Death [74]. A multispacer-typing detection of an Orientalis-like biotype in dental pulp specimens dating from the 5th to the 14th century established that the Justinian strains possess the *glpD* gene, while the strains from the medieval era, specifically the Orientalis biotype associated with the Black Death and later outbreaks, have the deletion in the *glpD* gene [78]. Furthermore, the absence of certain genetic markers, such as the *pla* TT insertion, characteristic of the Orientalis biotype [40], in medieval specimens further supported the presence of an Orientalis-like biotype during the Black Death. In summary, these findings collectively indicate that the Orientalis biotype played a role in all three pandemics, challenging the previously held notion that each pandemic was caused by a distinct biotype (Antiqua, Medievalis, and Orientalis) [51]. Consequently, the earlier conviction that the Antiqua and Medievalis biotypes were responsible for ancient epidemics must be reconsidered [73].

## 4. Microorganism Profile

### 4.1. Discovery of the Bubonic Plague Agent

The discovery of the bacterium cause of bubonic plague was made during the Hong Kong epidemic in 1894, when the French–Swiss bacteriologist Alexandre Yersin from the Pasteur Institute, and his Japanese colleague Kitasato Shibasaburo, independently and within a few days of each other, announced the isolation of the bacterium responsible for the Third Bubonic Plague Pandemic [14]. Kitasato initially claimed the discovery, but Yersin’s bacillus more closely resembles *Yersinia pestis* as it is currently described. On the contrary, the isolate described by Kitasato was distinct and probably due to a contaminating pneumococcus.

Effectively, although Kitasato had discovered the bacteria a few days earlier, controversy persisted within the scientific community for many years regarding whether he had indeed identified the correct bacterium. The discordance arose due to “vague and contradictory” reports from Kitasato’s laboratory. In blood samples, he described them as round pairs cocci, but in tissues, he claimed they appeared rod-shaped. On the other side, Yersin’s report characterized the bacteria as Gram-negative rod-shaped microbes. An independent retrospective analysis conducted in 1976 on the organism discovered by Kitasato helped clarify the issue [44]. While microbiologists conducting the analysis found it probable that Kitasato’s samples were contaminated with another bacterium, *Streptococcus pneumoniae*, there is “little doubt that Kitasato did isolate, study, and reasonably characterize the plague bacillus”, and he should, therefore, not be deprived of due credit.

Additionally, Yersin developed an antiserum against the recently discovered possible plague agent and, in 1896, used it to cure a plague patient [14,82,83]; later, he was also able to associate rats to plague, after *noting that rats were affected by plague during human epidemics*, although the role of fleas in plague transmission was independently discovered by Ogata and Simond, who during the Indian epidemic in 1897 demonstrated that fleas were the carriers of *Y. pestis* bacteria through experiments with infected rats [25,26,27]. These findings marked a significant advancement in the scientific comprehension of the disease, leading to subsequent progress in the development of treatments and vaccines [73].

Since its discovery, *Y. pestis* has suffered several nomenclature changes. Initially, the microorganism was named *Bacterium pestis* until 1900, *Bacillus pestis* until 1923, *Pasteurella pestis* (after Yersin’s mentor), and definitively, in 1970, was renamed as *Yersinia pestis* [83]. Formerly, it was taxonomically located in the *Enterobacteriaceae* family and in the *Yersinia* genus. Finally, in 2016, the *Yersinia* genus was included in the new family *Yersiniaceae* within the order *Enterobacterales* ord. nov., now formed of seven families: *Enterobacteriaceae, Erwiniaceae*, *Pectobacteriaceae*, *Yersiniaceae*, *Hafniaceae*, *Morganellaceae*, and *Budviciaceae* [84,85]. These families were proposed on the basis of phylogenetic analyses and the identification of conserved molecular characteristics that distinguish them from other families within the order *Enterobacterales*. The family *Yersiniaceae* contains eight validly published and correct genera names, including the type genera *Yersinia*, *Chania*, *Ewingella*, *Rahnella*, *Rouxiella*, *Samsonia*, and *Serratia*. Members of this family are motile, catalase-positive, and do not produce hydrogen disulfide. They are often associated with animals and can cause diseases such as urinary tract infections and plague.

The *Yersinia* genus is widely distributed in various natural environments and, as of November 2023, comprises 26 species with validly published and correct names [85]. The best-known human pathogens within the genus *Yersinia* are *Y. pestis*, *Y. enterocolitica*, and *Y. pseudotuberculosis* [55]. *Y. pestis* is a highly virulent pathogen that causes the potentially fatal systemic disease known as plague and stands out for its association with animal hosts, usually small mammals, mainly rodents. The transmission of plague to humans typically happens via flea bites, although it can also occur through direct contact with infected animals, consuming contaminated food, or inhaling aerosols [10]. *Y. enterocolitica* and *Y. pseudotuberculosis* are primarily enteric pathogens that are relatively uncommon and rarely cultured from blood, which can induce infections typically associated with the ingestion of contaminated food products or water, resulting in yersiniosis, a condition leading to global cases of gastroenteritis [55,86,87].

### 4.2. Pathogenesis and Virulence Factors

The pathogenesis of *Y. pestis* involves a complex interplay of virulence factors and immune subversion strategies. *Y. pestis* has evolved a number of virulence determinants that enable it to survive and cause disease in mammalian hosts and to persist in flea vectors. These determinants include various plasmids, proteins, and regulatory systems that contribute to its pathogenicity [14]. The factors responsible for virulence are complex and only partially understood, and while infectivity appears to depend on the presence of combined proteins, virulence appears to depend on other factors. Although chromosomally encoded virulence factors to cause disease are evidently important, plasmids play a crucial role in the virulence of *Y. pestis*. and require the three well-characterized virulence plasmids pCD1 (also known as pYV), pPCP1 (or pPla), and pMT1 (or pFra) [59].

The pPCP1 plasmid encodes for the plasminogen activator (*pla*) protease gene. The Pla protease is important for the invasion of the bacterium into host tissues and for the establishment of systemic infection [14]. It has also been shown that Pla is a significant virulence factor, responsible for the lung infection associated with *Y. pestis* but not with other *Yersinia* species [55], even though its absence does not prevent *Y. pestis* from causing pneumonic plague, as other factors may compensate for the lack of Pla activity [57]. The Pla protease mediates the degradation of complement components C3b and C5a, hindering opsonization and phagocytic migration. Additionally, it acts on fibrin clots, facilitating the rapid dissemination of *Y. pestis* [5].

The bacterial surface of *Y. pestis* contains two protein–lipoprotein complexes, the F1 (fraction 1) [59], and V or LcrV antigens [88,89], whose function is to contribute immune escape through preventing phagocytosis by macrophages and other phagocytic cells [90]. The genes (*caf*) for the F1 capsule are encoded by pMT1 [59]. The genes encoding for the low-calcium-response LcrV antigens are located on the pCD1 (or Lcr plasmid) in *Yersinia pestis* [91]. These antigens are produced by the bacterium at the normal human body temperature. Moreover, *Y. pestis* not only survives but also generates F1 and V antigens while residing within white blood cells like monocytes but not in neutrophils [92]. On the other side, the bacilli located in the gastrointestinal tract of fleas and rats lack V capsular antigens, making them susceptible to rapid ingestion and destruction by polymorphonuclear leukocytes. The virulence of the bacilli in the flea is concealed by the lower temperature (around 25 °C) at which they reproduce. When these bacilli are phagocytosed at 37 °C by monocytes (as opposed to granulocytes), they not only survive but also proliferate within the cells, subsequently emerging as fully virulent microorganisms equipped with antiphagocytic factors Fl and V [93].

A common characteristic of pathogenic *Y. pestis* is its ability to resist phagocytic killing [28], attributed to its ability to evade and suppress the host immune system, allowing it to proliferate and spread within the host. The T3SS mediates this property, injecting effector proteins into host cells to manipulate host processes that conduct the modulation of immune responses, facilitating infection. The Yops are a set of virulence determinants that are secreted by a type III secretion system. These proteins interfere with host cell signaling pathways and immune responses, aiding in the bacterium’s ability to resist phagocytosis and to suppress the immune response [94]. For example, YopM has been shown to inhibit platelet aggregation and is necessary for the virulence of *Y. pestis* in mice [95]. Upon interaction with phagocytic cells, the bacteria release proteins into the phagocyte that mediate the dephosphorylation of various proteins essential for phagocytosis (YopH gene product). Additionally, these proteins induce cytotoxic effects by disrupting actin filaments (attributed to the YopE gene product) and trigger apoptosis in macrophages (facilitated by the YopJ/P gene product) [96]. The T3SS also suppresses cytokine production, in turn diminishing the inflammatory immune response to infection. *Y. pestis* also possesses a T3SS called Ysc, which is activated upon contact with host phagocytes. The Ysc T3SS helps the bacteria evade the host immune system and establish a systemic infection, leading to septicaemic plague [55,97,98]. The pH 6 antigen is a protein that contributes to the adherence and invasion of *Y. pestis* and is expressed at the mammalian body temperature of 37 °C, which is indicative of its role during infection [99,100].

### 4.3. Adaptations of Yersinia pestis

The transmission of *Y. pestis* from fleas to mammals is facilitated by the formation of a biofilm in the flea’s digestive tract. This biofilm is crucial for the bacterium’s survival in the flea and for efficient transmission to mammalian hosts during a blood meal. Effectively, in contrast to other *Yersinia* species (*Y. enterocolitica* and *Y. pseudotuberculosis*), *Y. pestis* lacks an intestinal phase in its infection cycle, establishing a biofilm in the foregut of the rodent flea vector [55]. The bacterium in the flea employs two modes of transmission: early-phase or mass transmission and late-stage biofilm-dependent transmission. Between the key adaptations of *Y. pestis*, a blockage of the flea proventriculus forms a large mass of bacteria in the proventriculus, a sphincter-like structure that separates the flea’s stomach and midgut [56]. As a result, the bacteria are transmitted to the host, leading to the spread of the plague. Another important adaptation to its insect vector that *Y. pestis* has undergone is the ability to form biofilms in the flea’s midgut. This biofilm provides a protective environment for the bacteria, allowing them to survive and multiply within the flea. The colonization of the flea midgut is essential for the bacteria’s survival and subsequent transmission, and *Y. pestis* possesses specific factors that enable it, such as Ymt [71,101]. The biofilm formation and blockage of the proventriculus are essential for the effective transmission of *Y. pestis* from fleas to new hosts [72].

This bacterial biofilm formation is a complex process regulated by c-di-GMP and characterized by the secretion of an extracellular matrix (EPS). While little is known about the molecular mechanisms important for the initial step of biofilm development, the molecular and genetic mechanisms involved in the formation of bacterial biofilms in fleas have been identified. The hemin storage (*hms*) system involved in the formation of biofilms plays an important role [102]. In the case of *Y. pestis*, three key loss-of-function mutations have been identified that increase cyclic di-GMP-dependent biofilm formation in the flea foregut. In vitro, biofilm formation is mainly dependent on the diguanylate cyclase coded by *hmsT*, while in vivo, biofilm formation depends on the diguanylate cyclase coded by *hmsD* [86]. The biofilm matrix of many bacterial biofilms contains matrix-associated proteins and extracellular DNA. In *Y. pestis*, poly-β-1,6-N-acetyl-D-glucosamine is synthesized and exported by the *hmsHFRS* operon, which constitutes an important component of the biofilm matrix. The PhoP-PhoQ two-component regulatory system, which usually regulates virulence in several bacterial pathogens, in *Y. pestis* functions to alter the bacterial outer membrane in response to specific environmental stresses once induced in the flea and is essential for the complete formation of a consistent extracellular polysaccharide (EPS) [101,103]. The gene expression of the *hmsHFRS* operon and the Rcs signal transduction system is repressed by cyclic di-GMP [104].

Inactivation of EAL-domain phosphodiesterases (PDE) genes involving gene encoding and the RcsA component of the Rcs signal transduction system increases the levels of cyclic di-GMP, leading to enhanced biofilm formation [86,105]. Although several genes and regulatory systems that play a role in biofilm development in *Y. pestis* have been identified, the molecular mechanisms involved in several crucial aspects of biofilm formation are still not fully understood. Specifically, the molecular mechanisms surrounding initial bacterial attachment and the formation of biofilm auto aggregates are not yet clear.

Iron uptake systems are critical for *Y. pestis* survival and virulence, as iron is limited in the host environment. *Y. pestis* utilizes the yersiniabactin siderophore system to regulate iron assimilation [106]. *Y. pestis* can also uptake iron from heme-containing compounds through other transport systems that function at both ambient and body temperatures, i.e., the organism has the ability to absorb organic iron as a result of a siderophore-independent mechanism [14]. The complex pigmentation (*pgm*) locus [107] is involved in iron acquisition and storage, and the loss of this locus results in attenuated virulence. The Pgm+ phenotype is characterized by the adsorption of exogenous hemin, further facilitating iron assimilation, leading to the formation of pigmented colonies at 26 °C but not at 37 °C [107]. The iron uptake regulation in *Y. pestis* is controlled by the ferric uptake regulation (Fur) system [108]. Fur represses the expression of iron transporters to prevent the accumulation of toxic iron levels within the cell under iron abundance conditions. Under conditions of iron starvation, Fur does not bind iron and allows the expression of iron acquisition systems, enabling the bacterium to scavenge iron from the environment.

Moreover, *Y. pestis* has the ability to respond to low-calcium environments, which is linked to its virulence through a low-calcium response (LCR) system that is activated in the flea gut, where calcium levels are low. These proteins are encoded on plasmid pCD1 by the low-calcium-response (LCR) stimulon and play a crucial role in the bacteria’s ability to evade the flea’s immune system [14]. This system is essential for the expression of virulence factors, including the Yops that are secreted by a T3SS [72].

*Y. pestis* has been reported to survive and adapt to high-salt environments, such as salted lakes. This suggests that these high-salt environments contribute to the survival of *Y. pestis* in natural plague reservoirs [109]. The specific genetic and protein factors identified that are involved in regulating osmotic pressure and salt tolerance in *Y. pestis* include the upregulation of outer-membrane proteins, involving TolC efflux pump, OmpF porin, and Na+/H+ antiporters (*nhaA* and *nhaB*) [109]. The expression of specific proteins involved in energy production, cellular processes, signaling, and metabolism changed under high-salt conditions, contributing to adaptability and survival. As high-salt environments are common in arid regions globally, the findings of this study provide new insights into the biology and ecology of *Y. pestis* and the transmission patterns of the disease.

## 5. The Epidemiology of Plague

Plague is a highly contagious and lethal illness, affecting both wildlife and human populations as accidental hosts. *Y. pestis* can infect over 200 species of mammals, with rodents serving as the primary hosts, although carnivores can also be affected [56]. The enzootic cycle of *Y. pestis* in rodent populations, particularly in Asia, the Americas, and Africa, is the primary reservoir for the disease. Human outbreaks are often associated with spikes in rodent infections, which can lead to increased contact between infected fleas and humans [14].

### 5.1. The Urban and Sylvatic Plagues

There are two forms of *Y. pestis* infection: (1) urban plague (domestic), for which rats are the natural reservoirs, and (2) sylvatic plague (wild) [56,110]. The urban form of plague, representing the epidemic manifestation, persists within rat populations and spreads among rats or between rats and humans through infected fleas [3,7]. The close proximity of rat masses to humans facilitates transmission through bites from infected fleas, which acquire the infection during a blood meal from a bacteremic rat. After bacterial replication in the flea gut, the organisms can be transmitted to another rodent or to humans.

The sylvatic plague among small mammals, such as squirrels, prairie dogs, rabbits, and field rats, was first demonstrated in June, 1916, in San Mateo County by gross anatomical examinations of squirrels (*Citellus beecheyi*) [111,112]. Although direct human contact with these rodents is rare, flea bites during such encounters can transmit the plague. Consequently, these reservoirs may give rise to sporadic human cases when the mammals perish, and the flea vectors subsequently seek human hosts, representing a potential source for future epidemics. In both forms of plague, the initial transmissions result in bubonic plague [3]. Then, a bacteremia with *Y. pestis* may infect the lungs to cause pneumonic plague, which is transmitted in a human-to-human manner by the respiratory route without the involvement of fleas. Although the effective control of rats and better hygiene have eliminated the urban plague from most communities, in contrast, sylvatic plague is difficult or impossible to eliminate due to the widespread distribution of mammalian reservoirs and flea vectors [3,101,113]. *Y. pestis* induces fatal infections in animal reservoirs, resulting in cyclic patterns of human disease, corresponding to fluctuations in the number of infected hosts. Infections may also occur through the ingestion of contaminated animals or handling of their tissues [3]. Despite its high infectiousness, human-to-human transmission is rare unless the patient exhibits pulmonary involvement.

The main wildlife populations affected by plague include a wide variety of mammalian hosts, with rodents being the most common for *Y. pestis*. There are 351 species globally that can act as hosts, with 279 species of rodents identified as plague carriers [114]. Among these, around 70 species are identified as the primary reservoirs, predominantly located in regions corresponding to existing plague foci, including western North America, eastern South America, eastern Africa, Central Asia, and Southeast Asia [56,114]. Additionally, wild carnivores can also be affected by plague, as they become infected by eating infected prey (rodents) and can serve as indicators of infection among rodent populations [3]. In some cases, other animals such as squirrels and even bears have demonstrated antibodies against *Y. pestis* [115].

A study between 2005 and 2014 on plague in pumas in the Greater Yellowstone Ecosystem revealed that antibodies against *Yersinia pestis* were detected in 8 out of 17 (47%) pumas tested [116]. The organism itself (*Yersinia pestis*) was detected in 4 out of 11 (36%) pumas tested after necropsy, since they were found after they had already died. Neither puma sex nor age was significantly associated with *Y. pestis* exposure or mortality, although the sample size of 28 tested pumas was small. Plague is a significant source of mortality for local pumas, accounting for 6.6% of sub-adult and adult mortality. These findings show that plague is likely more prevalent than expected in the Greater Yellowstone Ecosystem [116]. According to the study, the presence of *Y. pestis* in pumas may increase the risk of zoonotic transmission to humans, which may be exposed to the bacteria by enzootic or epizootic hosts, by carnivores that eat these hosts or by fleas that are carried by any of these animals. Humans could be exposed to the plague through infected fleas seeking new hosts that are present on animal carcasses, through contact with blood or by handling diverse internal organs, depending on the specific type of plague [116]. While it is unlikely to get anyone sick because pumas usually do not go near humans, the study emphasizes that hunters and individuals interacting with pumas in this region should be informed and be aware about the potential for exposure. In the United States, sylvatic plague exists in about 15 Western states [14], and pumas may be a useful sentinel for potential risk of plague exposure to humans throughout the West [116].

### 5.2. Transmission of Plague

The disease cycle involves various animal species and fleas, and humans can become infected either directly or indirectly. The transmission of plague occurs primarily through the bite of a rat flea or other parasitic insects. The most common form results from the bite of the rat flea (*Xenopsylla cheopis*) or other parasitic insects such as the common louse (*Pediculus humanus*) or the human flea (*Pulex irritants*) [3]. Infection can also occur through close contact with infected wild and domestic animals, as well as through exposure to infected tissues and body fluids or inhalation of small respiratory particles between infected people or animals. Presently, in regions at risk, human infections frequently follow outbreaks in animals, and individuals may contract the infection through interactions with both wild and domestic animals or through flea bites [14]. When this occurs, the infected fleas, weakened and starving, abandon the dead rodents and seek blood from new hosts, including humans and their pets. Cats can become seriously ill in endemic areas and directly infect humans when they cough or sneeze bacteria-loaded particles. Dogs are less likely to suffer from the disease, but they can carry plague-infected fleas into the home and, through flea bites, people can be exposed [3].

Currently, sylvatic plague is endemic in parts of Africa, Asia, and the Americas, and outbreaks still occur. As the animal reservoir can vary depending on the region, this will be a key factor in both the risk of human transmission and the conditions under which it occurs. In some Asian countries, rodent meat and skins are consumed, potentially fostering close contact [3,14]. In summary, plague foci are maintained by enzootic hosts through interactions with fleas and contaminated soils [117]. The transmission of plague involves the transfer to epizootic hosts like prairie dogs and ground squirrels, ascending the food chain through smaller carnivores such as domestic cats and coyotes, and secondary predators like pumas. Pumas may also function as primary predators of epizootic hosts. Finally, human exposure may occur through contact with any of these animal groups [116].

## 6. Diagnosis, Treatment, and Prevention of Plague

### 6.1. The Clinical Diagnosis of Plague

Plague is a zoonotic infectious disease caused by *Y. pestis* that is primarily transmitted to humans through the bite of infected fleas, especially those that live on rats. The bacteria can enter the body through the skin, mouth, nose, or lungs. Plague occurs naturally in wildlife populations and can also be transmitted between humans. Plague should be considered for any patient displaying clinical signs of the disease and having a recent history of residing in or traveling to areas where plague is endemic (https://www.cdc.gov/plague/healthcare/clinicians.html, accessed on 19 December 2023).

Plague is diagnosed through a combination of clinical presentations and laboratory testing. The clinical presentations can include bubonic and pneumonic forms, septicemia, and, rarely, pharyngitis and meningitis [118]. Laboratory diagnosis of plague can be confirmed by bacteriological techniques, such as Gram examination and culture, serological examination, use of rapid diagnostic tests, or PCR [43,119,120,121].

#### 6.1.1. Clinical Presentations

The most common initial presentation of plague is bubonic plague, which affects the lymph nodes and is characterized by the development of painful buboes (inflammatory swelling of the lymph nodes) in the groin, axilla, or cervical nodes [67]. Symptoms initially include sudden onset of fever, chills, headaches, weakness, vomiting, and nausea, followed by the appearance of buboes (inflamed, tense, and painful due to replication of plague bacillus) [14,28,67]. The pain is often severe, leading to guarded movements and restricted mobility in the affected area. At advanced stages of the infection, the inflamed lymph nodes can turn into open sores filled with pus. The incubation period is no more than seven days after a person has been bitten by an infected flea [14,28]. Human-to-human transmission is rare (https://www.who.int/health-topics/plague, accessed on 30 December 2023). Even though bubonic plague usually responds quickly to appropriate antibiotic therapy (lowering mortality from 60% to 5%) [115], the lymph nodes remain enlarged and tender for 1 week [3]. Without treatment, *Y. pestis* can enter the bloodstream, leading to rapid dissemination and causing sepsis. In cases where the lungs are affected, pneumonia may develop.

The incubation period for septicemic plague is not clearly defined but is likely to occur within days of exposure. Septicemic plague can occur alone (absence of buboes) or secondarily to a bubonic form in combination with bubonic or pneumonic plague and results from a systemic infection with the bacteria. It can occur as a primary infection or can progress from bubonic or pneumonic plague. It involves the spread of the bacteria throughout the bloodstream, leading to septic shock and organ failure. Symptoms include fever, chills, weakness, abdominal pain, vomiting, diarrhea, and skin turning black and dying. It can be difficult to diagnose as it can present with non-specific symptoms that can be misinterpreted as sepsis [3,115]. Septicemic plague is fulminant and lethal in the absence of rapid supportive therapy that includes effective antibiotic treatment (mortality varies from 30 to 100%, according to the WHO).

Pneumonic or lung-based plague is the most virulent form and can occur when *Y. pestis* spreads to the lungs. Two clinical facts characterize this form, (1) a primary pneumonic plague, with 2 to 4 days of incubation following contact with a coughing patient, and (2) secondary pneumonic plague, which follows the dissemination of *Y. pestis* to the lungs during an episode of primary bubonic or septicemic plague [3]. It can be transmitted via droplets from an infected person or animal. It is highly contagious, and person-to-person transmission can occur through the air. The incubation period for pneumonic plague is typically 1 to 3 days, and symptoms include high fever, chills, cough, chest pain, difficulty breathing (dyspnea), and possible expulsion of bloody sputum [3]. If not diagnosed and treated early with specific antibiotic therapy, both pneumonic and septicemic plague can rapidly progress to fatal consequences, although recovery rates are high if detected and treated within 24 h of the onset of symptoms. The mortality rate in untreated patients with pneumonic plague comes close to 100%, but it fluctuates between 25 and 50% if appropriate treatment is administered within the next 24 h of the onset of symptoms [122,123].

Although the majority of plague patients commonly manifest a bubo, certain individuals may present with nonspecific symptoms. In instances of septicemic plague, individuals might present significant gastrointestinal symptoms, such as nausea, vomiting, diarrhea, and abdominal pain Additionally, there are less prevalent forms of plague, encompassing pharyngeal, meningeal, and cutaneous manifestations [3,124].

Definitively, a robust symptom surveillance system is necessary to identify potential patients as early as possible, and an important contribution comes from the study of which virulence factors specifically produce the clinical manifestations that plague presents in its primary clinical forms: bubonic, septicemic, and pneumonic. The F1 capsule protein inhibiting phagocytosis and allowing the bacteria to evade the immune system could contribute to the symptoms produced by the most common form, the bubonic plague, especially the appearance of painful, swollen lymph nodes (buboes), usually in the groin, armpit, or neck. This bulbo at the lymph nodes appears due to bacterial proliferation and inflammation, where bacteria are phagocyted but evade destruction, resulting in the characteristic necrosis [28]. The rapidly developing signs of sepsis in the septicemic plague seem to be driven by the plasminogen activator (Pla) protease, which contributes to the bacteremia in the host by degrading fibrin clots and other extracellular matrix components, facilitating dissemination in the bloodstream and leading to systemic infection. Also, the T3SS would play a critical role in the progression of septicemic plague, allowing the bacteria to survive and multiply within the bloodstream. Lastly, the clinical presentation of pneumonic plague includes rapid onset of fever, headache, weakness, and rapidly progressing pneumonia with symptoms, such as shortness of breath, chest pain, and a productive cough, which may be bloody or watery. The T3SS, which can disrupt normal cellular functions and promote the characteristic inflammation and tissue damage in the lungs, and Pla both cause the severe respiratory symptoms characteristic of pneumonic plague. Finally, the virulence factors of *Y. pestis* play a critical role in the pathogenesis of plague and are directly linked to its severity and progression. For this reason, their understanding is crucial for the development of effective treatments and preventive measures against this deadly disease. While the virulence factors of *Y. pestis* such as the T3SS and Pla protease are known to contribute to the clinical manifestations of plague, there are uncertainties regarding genetic susceptibility, changes in clinical presentation over time, and the impact of the evolutionary dynamics of the bacteria on disease presentation.

#### 6.1.2. Laboratory Testing

A confirmed diagnosis is obtained through bacteriological techniques, serological examination, use of rapid diagnostic tests, or PCR [67,79,119]. Healthcare providers should consider plague in any patient displaying clinical signs of the disease who has a recent history of residing in or traveling to areas where plague is endemic. Because of the rapid and fulminant progression of the disease, it is necessary to apply methods that allow for an immediate preliminary diagnosis. Evidently, samples containing *Y. pestis* must be handled with great care due to the risk of severe infections among laboratory personnel. The practices for specimen processing should be performed in a certified Class II Biosafety (BSL2) cabinet; this includes the detection of *Y. pestis* using immunological and nucleic acid-based methods [67]. A biosafety level 3 (BSL3) should be used for activities involving manipulations of cultures, which have potential for aerosol production, large volume, or high titer culture.

The standard method for diagnosing plague in a laboratory setting involves the isolation and identification of the plague pathogen from clinical samples (https://www.cdc.gov/plague/healthcare/clinicians.html, accessed on 19 December 2023). If there is a suspicion of plague, it is advisable to collect pre-treatment samples, if feasible; however, the initiation of treatment should not be postponed. The specimens suitable for diagnostic purposes should be collected from sites that allow for the isolation of bacteria based on the clinical presentation. These may include bronchial/tracheal aspirates (≥1 mL), whole blood (5–10 mL in EDTA), and/or inoculated blood culture bottles. Additionally, lymph node aspirates, aspirates or biopsies from the liver, spleen, bone marrow, lung, or bubo are considered appropriate. The bronchial/tracheal wash samples are recommended for suspected pneumonic plague cases. Throat specimens are not preferred for *Y. pestis* isolation due to the presence of numerous other bacteria that can mask its presence.

The pathogen can be cultivated on many routinely used media, including brain heart infusion broth, MacConkey agar, and sheep blood agar. The colonies formed at room temperature and 35–37 °C on the agar plate after a 48 h incubation are small (about 1 to 2 mm in diameter), with raised centers and a flat periphery, grey-white translucent, non-hemolytic in 24 h on sheep blood agar and chocolate agar [67]. Contrary to most pathogenic species, *Y. pestis* grows optimally at 26 to 28 °C (although incubation at 37 °C is necessary for F1 antigen production). After 48 h of growth, colonies have an average diameter of 1–2 mm, grey-white to slightly yellow opaque color that raises irregular, “fried egg” morphology or “hammered copper” shiny appearance (48–72 h). *Y. pestis* grows on MacConkey agar (48 h) as small, clear, or white non-lactose fermenter colonies. On cefsulodin-irgasan-novobiocin (CIN) agar, the colonies are colorless, developing pink centers. Growth in broth culture at 35–37 °C during 48 h is flocculent, producing structures resembling “stalactite” and clumps at the side and bottom of tubes. Routine blood cultures are a sensitive method for detecting plague, especially in later stages when bacteremia levels are high enough for organisms to be occasionally seen on blood smears. The culture of sputum is possible in severely ill patients with pneumonic plague, although blood cultures are usually positive at this stage.

The additional key characteristics for the identification of cultured microorganisms include lysis with specific bacteriophage at 22 to 25 °C and agglutination with specific antiserum and biochemical characteristics (to differentiate them from other yersinias). Thereby, isolates of *Y. pestis* are positive without gas production for glucose and mannitol fermentation and negative for the fermentation of lactose, sucrose, rhamnose, and adonitol. *Y. pestis* is catalase positive and negative for urease, oxidase, and indole. The microorganism is non-motile at 25 °C and 35–37 °C, being the only species of *Yersinia* which is non-motile at room temperature. Although included in most enteric automated identification systems, *Y. pestis* lacks biochemical activity in certain assays, and as a result, conventional biochemical identification systems may occasionally lead to misidentification with *Y. pseudotuberculosis* or other enterobacteria [10,125]. Even matrix-assisted laser desorption ionization–time-of-flight mass spectrometry (MALDI-TOF MS) identification systems may misidentify the cultured organism. Consequently, the identification of *Y. pestis* must be considered presumptive until reference laboratory confirmation.

*Y. pestis* may be identified microscopically by examination of Gram, Wright, Giemsa, or Wayson’s stained smears of peripheral blood, lymph node specimen, or sputum. The cells of *Y. pestis* appear as small pleomorphic Gram-negative rods (0.5–0.8 × 1–3 μm) by Gram staining [14]. The tendency to bipolar staining (resembling a “safety pin”) may be poor with Gram stain staining but more apparent with Giemsa or Wayson staining (methylene blue and carbofucsin) in tissue preparations, bubones, and, to a lesser extent, cultures. Newly isolated virulent strains produce an abundant capsule. Since an affected bubo typically contains numerous organisms, the microscopic and culture evaluations of lymph node aspirates are useful. The smears of sputum or aspirated lymph node fluid should be stained using the Gram method and with Wayson’s reagent or methylene blue to observe bipolar staining. During pneumonic plague outbreaks, the use of fluorescent antibodies is highly valuable for the rapid identification of *Y. pestis* in sputum. Finally, in those cases of viable but uncultivable microorganisms, direct detection methods such as direct fluorescent antibody (DFA) or PCR may provide presence evidence of *Y. pestis* in lymphoid, spleen, lung, liver tissue, or bone marrow samples.

Solid media containing Ab against the F1 antigen have been used to identify the colonies of *Y. pestis* in mixed cultures, since in such plates, the treatment with chloroform vapor releases the Ag, and a precipitation ring is formed around each colony. Additionally, the F1 antigen typically serves as a common target for the immune detection of *Y. pestis* through traditional methods, such as passive hemagglutination and the inhibition of the F1 antigen hemagglutination tests, which are routinely used for the identification of the F1 antigen [126]. Nevertheless, the quantitative detection of F1 antibody or F1 antigen has been described through direct fluorescent antibody testing and enzyme-linked immunosorbent assays [127]. Also, *Y. pestis* can be detected by PCR through the F1 antigen gene (*caf1*), *pla* gene, or chromosomal fragments like fragment 3a [73,128]. However, recent findings indicate that the *pla* gene and chromosomal fragment targets are untrustworthy for *Y. pestis* detection [67]. A critical concern impacting *Y. pestis* diagnostics relying on the *pla* gene detection is the presence of Pla-lacking strains in certain regions, where such strains are not unusual [57]. Relying, in these areas, solely on Pla-based *Y. pestis* detection systems or in the face of a potential bioterrorism episode poses a certain risk.

In regions lacking an equipped laboratory for the bacterial isolation, identification, and immunological or molecular detection described above, point-of-care testing (POCT) has been demonstrated to be beneficial [67], e.g., rapid on-site detection is facilitated by the utilization of immunochromatographic assays (ICAs), a simple and rapid test with high sensitivity and specificity [119]. The colloidal gold-based ICA addresses the immediate requirement for on-site detection in remote locations; nevertheless, its execution needs skilled professionals to guarantee result accuracy [67,119]. A novel ICA utilizing up-converting phosphor technology, i.e., using materials capable of absorbing lower-energy and emitting higher-energy photons, has been created and offers advantages, particularly in scenarios where precise quantification and reliable results are essential [67]. Portable, real-time quantitative PCR thermocyclers can be used for the on-site detection of plague pathogens [129].

Serological tests have significance in a retrospective context. If cultures produce negative results while there is still suspicion of plague, serological tests can be employed to validate the diagnosis. For this purpose, it is advisable to collect one serum specimen as early as possible during the illness, followed by a convalescent sample taken 4–6 weeks or more after the onset of the disease.

### 6.2. Treatment of Plague

Plague is a dangerous disease that spreads rapidly and can cause high mortality if not treated promptly. For this reason, early diagnosis and prompt initiation of treatment are crucial to reduce the mortality rate associated with plague. The preferred treatment for plague involves the administration of antibiotics, while a vaccine is available but is not widely used. The recommended antibiotic options include aminoglycosides like gentamicin or streptomycin or, alternatively, fluoroquinolones or doxycycline. The treatment recommendations for patients who are elderly or immunocompromised do not differ from those for adults, but clinicians should monitor these patients carefully and adjust antimicrobials accordingly [3,10,67]. The administration of effective antibiotics and antishock therapies should not be delayed for more than 24 h. The choice to start antibiotic treatment for plague should rely on assessing clinical indications, symptoms, and a detailed patient history. Factors such as a recent flea bite, exposure to rodent-populated areas, or contact with a sick or deceased animal increase risk for plague in regions where the disease is prevalent. While specialized laboratory tests can later confirm the diagnosis, it is essential not to postpone or withhold treatment while waiting for these test results.

While the majority of *Y. pestis* isolates globally exhibit sensitivity to streptomycin, an isolated multi-drug-resistant (MDR) strain was identified in Madagascar [122,123]. Providentially, this MDR strain has not reemerged naturally in this region or elsewhere. In this sense, there is concern regarding the potential utilization of this MDR strain (resistant to streptomycin, chloramphenicol, ampicillin, spectinomycin, kanamycin, tetracycline, sulfonamides, and minocycline), for instance, in acts of bioterrorism [124]. Regarding biosafety, *Y. pestis* is classified as a bioterrorism agent of category A (a carefully selected agent with bioterrorism potential). Consequently, such MDR strains could be tempting for bioterrorists pursuing a deliberate release, i.e., a bioterrorism attack [28,67,124,130]. Effectively, these topics have been increasing in interest, and simple literature searches [28] on MEDLINE/PUBMED (19 Dezember 2023) using a search string including ((((Deliberate Release) OR Intentional release) OR Biowarfare) OR Bioterrorism) OR Terrorism, provided 27,802 results and the search for (((Plague) OR *Y. Pestis*)) AND (((((Deliberate Release) OR Intentional release) OR Biowarfare) OR Bioterrorism) OR Terrorism) reached 578 results. In the event of infection with these strains, e.g., in a large-scale plague outbreak or bioterrorism attack setting, caution is advised in choosing effective antibiotics, avoiding the issues mentioned above. Although oral doxycycline and ciprofloxacin are recommended to treat the plague for both adult and child patients [67], the treatment of those infected with a wide range of antibiotics (for example, trimethoprim- sulfamethoxazole) should not be discarded, and it has successfully been used in a single case of MDR plague [28,131].

Definitively, early treatment is the key to recovery and can reduce the risk of complications and death. Patients with bubonic or septicemic plague generally do not need to be hospitalized and can be treated with antibiotics in an outpatient setting. However, patients with pneumonic plague require hospitalization for isolation and treatment with antibiotics. Treatment for pneumonic plague should always be administered under strict medical supervision.

### 6.3. Prevention

The prevention of plague includes measures to reduce the risk of exposure to infected fleas or their hosts, such as rats. Control measures include rodent control, use of insecticides, and minimizing exposure to rodents and their fleas. Travelers to endemic areas should take precautions to avoid flea bites, such as wearing protective clothing and using insect repellent. In areas with high incidence of pneumonic plague, protective masks can be worn to reduce the risk of transmission. Controlling the spread of the disease involves reducing opportunities for human–flea–human contact. It also requires improved environmental management, including reducing the rat and flea populations and maintaining good hygiene practices. Improving public awareness of the risks of plague and its modes of transmission is also crucial.

The measures to mitigate the potential spread of plague in wildlife populations and prevent human exposure involve a comprehensive approach [3]. This includes the continuous monitoring of wildlife populations for signs of plague and conducting tests for *Y. pestis* in animals. Educational initiatives aimed at individuals, such as hunters, trappers, taxidermists, biologists, and managers, involved in handling live and deceased animals, informing them about the potential transmission of plague from wildlife to humans. Furthermore, encouraging the use of personal protective equipment when handling animals that may be infected with *Y. pestis* proves crucial. Promoting the implementation of flea control measures for both pets and domestic animals contributes to overall prevention strategies. In areas where fleas may be present, the use of insect repellent is strongly recommended. Additionally, individuals are advised to avoid contact with sick or dead animals, particularly rodents and carnivores. Administering antibiotics is recommended for those exposed to *Y. pestis* or presenting symptoms of plague. Lastly, ensuring people report sick or dead animals to state and provincial wildlife agencies could enhance surveillance and response mechanisms.

## 7. Vaccine

The development of a plague vaccine in 1897 played a key role in mitigating the impact, particularly in India and subsequent outbreaks in the 1900s [132]. The prevalence of plague cases markedly diminished throughout the twentieth century, attributable to advancements in medicine, sanitation, and the eradication of natural reservoirs [14]. Presently, a very small number of plague cases persist, with 95 percent occurring in sub-Saharan Africa and Madagascar [123,133].

The development of vaccines against plague has been an ongoing effort since the identification of the causative organism, *Y. pestis*. Historically, the USP vaccine, which was composed of formaldehyde-killed *Y. pestis* 195/P, was used in the West for several decades. This vaccine primarily elicited immunity through the expression of the capsular antigen F1, but it was reactogenic, provided only short-term immunity, and did not offer robust protection against pulmonary exposure to *Y. pestis* [28,134]. Live attenuated vaccines (LPVs) have also been developed, with the breakthrough LPV introduced by Girard and Robic using the attenuated variant EV76 [135,136]. This vaccine was widely used in the USSR and is still in use in some former Soviet Union countries to immunize individuals in plague-endemic areas. However, it requires annual boosters due to its short-term protection, and the underlying reasons for this are not fully understood.

Recent advancements in plague vaccine development include oral vaccination with *Y. pseudotuberculosis*, which shares genetic similarities with *Y. pestis*, bacteriophage vaccines, and a recombinant vaccine targeting the F1 capsular antigen and the low-calcium-response V antigen (LcrV) of *Y. pestis* [28]. The latter has been granted “orphan drug status” by the FDA for marketing after successfully completing phase 2 trials [137]. However, it does not protect against F1-negative strains of plague, which could potentially be used as a bioweapon. Subunit vaccines using recombinant antigens, particularly LcrV alone or in combination with F1, have shown promise in various formulations, including with adjuvants, in micro- and nanoparticles, and in viral and bacterial vectors [134]. These vaccines have been tested in animal models, such as mice, rats, guinea pigs, and non-human primates, with LcrV providing most of the protection and F1 enhancing the immunity level [28].

## 8. Things to Do in Plague Research

Plague is an ancient and persistent disease that still poses a significant threat to public health, and controversies and gaps in knowledge persist across different unresolved aspects that include the evolutionary dynamics, ecological interactions, molecular mechanisms, genetic diversity, and the impact of climate and environmental factors in relation to *Y. pestis*, urging concerted efforts for a more complete understanding of this pathogen [56].

The origins and spread of historical pandemics, as shown, remain unclear, with the lack of ancient genomes from outside Eurasia affecting our understanding of the historical origins and spread of plague. The complexities of *Y. pestis* infection and transmission, history, and epidemiology are always necessary to gain a comprehensive understanding of this ancient and persistent disease. Reconciling the source of a pandemic cannot be accomplished by a single research discipline, and interdisciplinary approaches to pandemic sources need to be applied. Recovering evidence from ancient *Y. pestis* genomes from Africa may provide insights into the sources of plague and their persistence over time. Combining biological evidence from these genomes with historical and archaeological contexts is necessary to fully understand, for example, to confirm challenging diagnosis of historical plague outbreaks, such as the medieval Black Death or the Athenian Great Plague [8], or the study of teeth collected from victims to confirm whether septicemic plague was specifically diagnosed before the end of the 16th century in France [38]. Moreover, although it is known that *Y. pestis* acquired certain plasmids and inactivated numerous functional genes during its evolution from *Y. pseudotuberculosis*, the order of these events and their fitness advantages are not clear. Additionally, the reasons for distinct animal virulence across different phylogroups of *Y. pestis* are not fully explained, except for a few clues related to fine genomic changes.

The genetic and phenotypic diversity of *Y. pestis* strains that cause disease in different regions of the world remains an important issue, with the organism being introduced to some parts of the world recently, i.e., *Y. pestis* strains that cause disease in the Americas are limited because the organism was introduced to this part of the world relatively recently (just over 100 years ago) [72]. Understanding the impact of variation in strains from natural foci, for example, in the former Soviet Union (FSU) and Asia, is important as these regions have had much longer periods of enzootic residence [38,72].

The role of how different vectors transmit the disease is also not fully understood and further investigation is necessary. For example, the role of *Cimex lectularius* (the bed bugs), a species of *Cimicidae*, as an effective interhuman vector of plague should be further investigated [3]. In this context, further investigation is needed to improve understanding of how *Y. pestis* senses and adapts to temperature shifts during its transition from flea (or other vector) to mammalian hosts. This includes a better knowledge of how *Y. pestis* survives in host innate immune cells during the early stages of infection, the mechanisms behind its substantial growth in host blood, which is crucial for flea transmission, and its effective immuno-suppression capabilities.

From an ecological and evolutionary point of view, the genetic diversity inherent in *Y. pestis* evidently plays a pivotal role in shaping the distribution and ecology of the disease, although how it contributes is not fully understood. But, also, the driving forces shaping ancestry and the detailed evolutionary dynamics of *Y. pestis* infection and its complex network of transmission, maintenance, and persistence require new insights. Additionally, although related, the intricate ecological interactions between *Y. pestis*, hosts, and the natural environment form a cornerstone for the long-term survival and sustained natural plague foci that remain enigmatic and necessitate deeper comprehension, especially concerning those molecular mechanisms and genetic variations governing the complex relationships among *Y. pestis* and niche factors, including the influence of climate.

Other aspects demanding constant analysis and advancement in knowledge include the evolving landscape of medical countermeasures, multidrug resistance (i.e., elucidation of resistance mechanisms and effective counterstrategies), biosafety regulations, and laboratory safety practices, emphasizing the risk of laboratory-acquired infections and the need for global and robust standardized containment practices when working with *Y. pestis*. In summary, despite not being an eradicated disease, plague is often perceived as a historical curiosity rather than an ongoing threat. Nevertheless, plague remains one of the most feared infectious diseases, and multidisciplinary approaches to plague research combining omics, archaeology, history, and epidemiology would be beneficial and necessary to gain a more comprehensive understanding of all these pending issues.

## 9. Conclusions

Based on the comprehensive analysis presented, it is evident that the plague remains one of the deadliest infectious diseases known to humanity. The extensive historical accounts of its desolating effects and the genetic characterization together with the genomic analysis of its spread offer invaluable insights into the epidemiology of modern epidemics. The molecular analysis of *Y. pestis* evolution from its ancient origins as well as its adaptation to flea-borne transmission, and its impact on human and wild populations, significantly advances our understanding of its genetic, antibiotic resistance, and antigenic diversity. The genetic richness of the bacteria, obtained from diversity, seems to explain their adaptive capacity and resilience, even in adverse circumstances. The findings demonstrate the essential role of bioinformatics in the analysis and interpretation of genetic information, especially from ancient DNA, including the unique misincorporation characteristics that help in the authentication of ancient origin. These tools, including automated pipelines, offer invaluable assistance to researchers in identifying specific *Y. pestis* lineages. Finally, it is evident that plague, but also other zoonotic diseases, requires continuous preventive surveillance to mitigate any future outbreaks. This can be achieved by improving molecular and genetic screening of animal populations, identifying ecological and social determinants of outbreaks, increasing interdisciplinary collaborations among scientists and public healthcare providers and continued research into the characterization, diagnosis, and treatment of these diseases.

## Data Availability

No new data were created.
